# PatientDataChain: A Blockchain-Based Approach to Integrate Personal Health Records

**DOI:** 10.3390/s20226538

**Published:** 2020-11-16

**Authors:** Alexandra Cernian, Bogdan Tiganoaia, Ioan Sacala, Adrian Pavel, Alin Iftemi

**Affiliations:** 1 Faculty of Automatic Control and Computers, University Politehnica of Bucharest, 060042 Bucharest, Romania; ioan.sacala@upb.ro; 2 Faculty of Entrepreneurship, Business Engineering and Management, University Politehnica of Bucharest, 060042 Bucharest, Romania; bogdan.tiganoaia@upb.ro; 3 Setrio Soft SRL, 062204 Bucharest, Romania; adrian.pavel@setrio.ro; 4 Modex Ltd., 031625 Bucharest, Romania; alin@modex.tech

**Keywords:** electronic health records (EHR), personal health records (PHR), blockchain, Modex blockchain database (BCDB), healthcare sensors

## Abstract

Currently there is not a single trusted infrastructure used for the exchange and storage of medical data along the healthcare value chain and, thus, there is no platform used for monitoring patients’ traceability within the entire healthcare chain. This situation leads to difficult communication and increased procedural costs, and thus it limits healthcare players from developing a better understanding and know-how of patients’ traceability that could further boost innovation and development of the best-fitted health services. PatientDataChain blockchain-based technology is a novel approach, based on a decentralized healthcare infrastructure that incorporates a trust layer in the healthcare value chain. Our aim was to provide an integrated vision based on interoperability principles, that relies on the usage of specific sensors from various wearable devices, allowing us to collect specific data from patients’ medical records. Interconnecting different healthcare providers, the collected data is integrated into a unitary personal health records (PHR) system, where the patient is the owner of his/her data. The decentralized nature of PatientDataChain, based on blockchain technology, leveraged the proper context to create a novel and improved data-sharing and exchange system, which is secure, flexible, and reliable. This approach brings increased benefits to data confidentiality and privacy, while providing secure access to patient medical records. This paper presents the design, implementation, and experimental validation of our proposed system, called PatientDataChain. The original contributions of our paper include the definition of the concept of unifying the entire healthcare value chain, the design of the architectural model of the system, the development of the system components, as well as the validation through a proof of concept (PoC) conducted with a medical clinic from Bucharest, using a dataset of 100 patients and over 1000 transactions. The proof of concept demonstrated the feasibility of the model in integrating the personal health records from heterogeneous sources (healthcare systems and sensors) in a unified, decentralized PHR system, with enhanced data exchange among healthcare players.

## 1. Introduction

Although technology is constantly evolving and we can now benefit from big data, machine learning, and enhanced interoperability capabilities, patient data is highly fragmented, as each healthcare provider holds its own medical health records [1]. This means that doctors do not have access at every medical appointment to the entire medical history of the patient and previous exams, diagnosis, and treatments, whereas patients have to go over their medical history each time, wasting time and losing accuracy.

### 1.1. Context

The modern medical world is quickly advancing from the technological perspective, implementing patient records digital management as well as the usage of innovative, data-driven equipment. Nowadays, the healthcare sector uses electronic health records (EHRs) [2] and personal health records (PHRs) [3] to store medical data. EHRs and PHRs aim to facilitate and increase access to patients’ data, and consequently to reduce medical errors and their associated costs and losses [1,2].

#### 1.1.1. Electronic Health Records

According to ISO/TR 14639, the electronic health record (EHR) is ‘‘information relevant to the wellness, health, and healthcare of an individual, in computer processable form and represented according to a standardized information model” [4]. An EHR is a standardized model for representing medical data [5]. EHRs are operated by medical institutions, and data is entered by clinicians. The patient has no access or control over his health records. EHRs store a patient’s demographics, medical history, diagnoses, medications, treatment plans, immunization dates, allergies, radiology images, and laboratory and test results [3]. Their purpose is to gather data from medical stakeholders, and leverage electronic collaboration and information sharing among medical institutions, while the patient is only a passive actor.

Their main advantage is the fact that they enable the integration of data between healthcare providers [6]. Other important clinical benefits include a reduction in medical errors and of medication errors, improving disease management, and improved quality of care [7]. However, the main limitation of EHR systems is related to interoperability [8]. Other limitations are related to data security, data exchange between healthcare organizations, or to the fact that they do not integrate any data about a patient’s lifestyle and wellness [5].

#### 1.1.2. Personal Health Records

A personal health record (PHR) is an electronic record that allows patients to manage and access their medical data [9]. The fact that it is the patient in charge of maintaining the data is considered the main advantage over EHRs [10]. Thus, the patient can also introduce details like weight, blood sugar, and more, as well as data withdrawn from various sensors that monitor their health state. PHRs’ purpose is to provide a comprehensive overview of the patient’s medical history, containing data entered by the patient, lab results, as well as data from devices such as wearables sensors or collected from a smartphone [11].

The main barriers in the adoption of PHRs are related to their difficult usability and low level of adoption—as patients are responsible for maintaining their own health records—as well as a lack of integration with existing electronic health records systems used by medical institutions [9]. Other challenges are related to data protection issues and third-party access permissions, such as from clinicians, to operate the data [3].

#### 1.1.3. Interoperability Standards in Healthcare

Standards define a shared vocabulary and a shared set of expectations that make it possible for systems and/or devices to interoperate. Standards enable physicians, laboratories, clinics, pharmacies, and patients to exchange data regardless of application or business supplier, in order to seamlessly digest information about a person, and enhance overall communication and delivery of healthcare [12]. In order to clarify the types of health data standards available for use, these standards are grouped into the following basic categories: vocabulary/terminology, content, transport, safety and protection, and identifiers [12]. Table 1 provides an overview of the main standards in each category:

#### 1.1.4. Wearables

Wearable sensors are now influencing healthcare and medicine by allowing remote health monitoring, analysis of the healthcare condition, and prediction of health events [22]. Wearables, as well as health apps, have grown in popularity in recent years [23] Besides monitoring physical exercise, heart rate, sleep, or calories, they are also able to support the management of chronically ill patients suffering from specific diseases such as Parkinson’s disease, neurocognitive disorders, or diabetes [24].

Wearable devices, such as health tracking devices or fitness trackers, generate impressive amounts of real-time data about the patients’ healthcare condition. Thus, integrating these data into the patients’ personal health records can improve the patient’s medical history, increase accuracy and support the doctors in identifying any change in patients’ health conditions and possible triggers, in order to reach a quicker diagnosis [25]. There is also an increasing number of devices that can be found on the market for tracking healthcare conditions, such as: smartphones, sports and fitness trackers (e.g., Apple, Fitbit, Garmin), sleep trackers and wearable monitors, smart glasses and head-mounted displays, electronic skin patches, wearable health alert and monitoring devices, minimally-invasive CGM (Continuous Glucose Monitoring) sensors, non-invasive CGM (Continuous Glucose Monitoring) sensors, ECG (Electrocardiogram) sensors, PPG (Photoplethysmogram) sensors, pregnancy and newborn monitoring, wearable temperature monitoring, hydration sensors, wearable sweat sensors (medical and sports), wearable drug delivery, smart footwear, smart contact lenses, wearable exoskeletons, medical hearables, or smart clothing [12,26].

The latest trends in wearable devices and sensors is combining them with AI and machine learning in order to make predictions and detect medical issues in a timely manner. The list below, although not exhaustive, summarizes some of the latest developments in this field.

- AI-based wearable devices to monitor healthcare at home

The AI-based wearable device developed by Current Health [27], which measures several vital signs, such as respiratory rate, oxygen levels, pulse, blood pressure, and body temperature, has recently received approval to be used at home by patients. The platform uses machine learning algorithms to analyze the data in order to predict and detect medical issues and alert providers to high-risk patients, so patients can receive healthcare in a timely manner.

Current research uses artificial intelligence to classify and detect specific cardiovascular diseases using a wearable wrist biosensor. One of the latest developments consists of a wearable sensor device using machine learning algorithms to detect hypertrophic cardiomyopathy [28].

Irregular and abnormal changes in body temperature can indicate some severe diseases. It is therefore necessary, under these conditions, to monitor body temperature in real time, with high accuracy. Health-monitoring temperature sensors require high resolution, high precision, high sensitivity, and wide detection range, but also biocompatibility and mechanical flexibility [29].

- Gait analysis data from wearables to predict Alzheimer’s and Parkinson’s diseases

Sensors-based gait analysis is currently being used as a method to predict Alzheimer’s disease [30] and Parkinson’s disease [31]. The walking patterns can be analyzed and classified through clinical assessment, by monitoring the patient’s walk. Modern approaches use wearable devices, trackers, even different contact sensors placed in shoes or socks to monitor walking parameters and provide an early prediction for Alzheimer’s and Parkinson’s diseases risk and monitoring.

- Biomolecule recording

Recent research has been conducted to integrate biochemical sensors (devices able to convert a chemical or biological quantity into an electrical signal) into a platform for noninvasive personal health monitoring at molecular level. These sensors have been used to monitor human cancer molecules and protein markers in real time [32].

The market drivers that favor the adoption of wearable devices in healthcare are related both to patients and physicians. There is a rising population of technology users on the patient side, increasing health and wellness popularity, and an increased demand for patient-centered treatment. The patterns in rising demand for wearables from the physician side include increased interest in big data and personalized, preventive care [33].

There are also several barriers related to this market. On the patient side, the high cost of wearables (usually over $150), the complicated reimbursement process, as well as data privacy and security issues. Owing to a perception of low data quality and a lack of scientific evidence that confirms the results, doctors are also hesitant [23].

#### 1.1.5. Blockchain

Blockchain is a distributed ledger technology (DLT), which securely records information across a peer-to-peer network, in a growing chain of immutable blocks linked by cryptographic hashes [34]. A distributed ledger is a form of decentralized database of transactions, that is shared and synchronized across multiple nodes. Each node owns an identical copy of the record, and records are automatically updated when new data are inserted.

A key concept behind blockchain is consensus. For new data to be added in the blockchain, all participants can see it and must reach consensus in order to accept or reject it. Once approved, data is stored into the ledger as a collection of “blocks” and cannot be altered. This is one of the best benefits of ledger technology. Another remarkable feature introduced with blockchain is smart contracts [34]—self-executing agreements based on blockchain technology—that automatically trigger actions or payments once conditions are met. As future trends, they will use real-time information, like GPS data, to trigger events, such as a transfer of ownership and funds. Moreover, blockchain is characterized by the following features [34]:

- Decentralization: unlike traditional database systems, which store information on a central server, blockchain removes this single point of failure issue through decentralization. Data is hosted and managed though consensus by all the parties involved in the business flow, without having a single point of control.
- Distributed and scalable: for a distributed ledger configuration, the backend is a network of computers, each storing applications and data. This distribution guarantees availability and fast access to the system.
- Security and transparency: the entire blockchain concept emerged from the need for a secure and stable framework, thus data protection, security, and cryptography are core features of this technology.
- Data immutability and integrity: the decentralized and distributed architecture ensure data immutability and integrity, given that data cannot be altered unless all parties in the blockchain reach consensus, increasing trust between users.

The technology is still new and under validation, but it has a huge potential in disrupting business models and shifting them from a centralized to a decentralized approach. The top five industries that are currently adopting blockchain solutions are: financial, healthcare, logistics/supply chain, legal, governmental, and oil and gas industries.

### 1.2. The Problem

Healthcare Information Technology (HIT) is constantly evolving, and the market is estimated to reach $297 billion by 2022 [1]. However, the main issue HIT is still facing is the fact that there still is no unified viewpoint of the entire patient’s health data. Each healthcare provider keeps its own health records and, in many cases, they do not share the data with the patient or with other medical systems. Moreover, healthcare data come from various sources, such as medical and treatment history, investigations, as well as health data collected from sensors that monitor the patient’s health or wearable devices [35].

In this context, the following four problems are longstanding in the healthcare field:

1. Lack of interoperability and difficulties in healthcare data exchange [36]. An essential issue now is the fact that hospitals and medical service providers are not interoperable. Consequently, medical health records are disparate, fragmented, and difficult to exchange. Thus, a patient ends up having their medical records stored in different locations: medical investigations are stored in the providers’ databases, medication data is stored locally with various pharmacies, etc. This lack of interoperability makes patients and providers face significant hurdles in initiating data retrieval and sharing. Thus, it becomes mandatory in the current context, where the volume of medical data generated is rapidly increasing, to define a common standard to integrate the patients’ healthcare records from various sources and enhance data exchange across the entire value chain.
2. Difficulties in ensuring data confidentiality and privacy, while maintaining secure access to patient records. At present, confidential medical records lack a protected structure, leading to serious data breaches. The Office for Civil Rights (OCR) of the Department of Health and Human Services (USA) issued reports of several data breaches in 2018 that culminated in the disclosure of 13 million total health records [37]. To become fully interoperable, healthcare data needs to be shared between healthcare stakeholders, while also protecting sensitive information and securing access only to authorized users. Data sharing and exchange is a great opportunity for improving the healthcare ecosystem on the road to interoperability, but also one of the biggest privacy challenges.
3. Data ownership. Another problem in healthcare today is related to data ownership, namely the fact that patients are unable to have complete control of their own medical records. This aspect’s importance increases with the rise of personalized medicine and wearables [38]. In the data-sharing context, one aspect that becomes essential is the patient’s consent to make this a transparent process [39]. Thus, for reaching a fully integrated and interoperable healthcare system, an important requirement is to grant the patient ownership of their data, which will enable them to decide who has permission to access their data and with what privileges [13]. This would help create a flexible and secure health records management. As a matter of fact, a common problem with all the existing systems is insufficient security controls for users accessing or sharing files [40], as there are major considerations concerning user privacy and therefore limitations of dominant access to personal information [41].
4. Patient data overload. Health Information Technology (HIT) has evolved, but still lacks a unified viewpoint over the entire patient’s health history [42,43]. One of the new approaches on personal health records focus on integrating healthcare data collected from various sensors, trackers, and wearable devices, such as fitness trackers, smartwatches, or devices that monitor healthcare parameters (heart rate, glucose level etc.) [44]. A challenge that occurs in this context is the huge amount of data that is produced, that need to be processed, analyzed and stored. Thus, new storage and processing solutions must be found [45]. In addition, a strong user interface (UI) becomes relevant in presenting the information to healthcare providers in an attractive and easy-to-interpret manner. Consequently, there is an increasing trend within healthcare organizations to focus on user experience, and engage UI designers in the process to address this need [46].

In view of the abovementioned concerns, our research objective was to develop a distributed and interoperable PHR system, built over blockchain technology, in order to provide a single, interoperable version of the medical health records of patients, that can be shared with healthcare providers, across the medical value chain.

## 2. Related Work

Our analysis of the state of the art followed two main directions: (1) distributed PHR systems and (2) blockchain in healthcare.

### 2.1. Distributed PHR Models

- The uPHR (Ubiquitous PHR framework) model [47] is an event-based distributed model. The model implements the standards HL7, CCR, and CEN 13606. Limitations: does not focus on security and data privacy.
- The HealthVault [48] is a web-based, client-server, PHR for keeping health and fitness records. It is compliant with HL7. Limitations: proprietary solution.
- The DEPR (Distributed Electronic Patient Records) model [49] is a distributed model based on the OpenEMR system, compliant with several standards. Limitations: does not focus on security or data privacy.
- My HealtheVet [50] is an online platform for sharing health information. It is designed on a distributed event-based model, and the interoperability model and security policies are based on the HL7 standard.
- The CF (Conceptual Framework) model [51] is a framework implementing a distributed mechanism for wearable health systems. It is compliant with the HIPAA standard for security and privacy. Limitations: does not focus on interoperability.
- The SNOW project [52] is a decentralized medical data processing system, based on the openEHR standard.

### 2.2. Blockchain in Healthcare

Using blockchain in healthcare has huge potential to address several of the current health system issues, such as immutable storage and distribution of patients’ medical records, thus providing access to more stakeholders involved in the medical process and offering the guarantee of data protection and security [53,54]. It can provide a novel approach to data ownership and permission-based access through the medical stakeholders’ value chain. Blockchain technology in healthcare can make the transition from traditional interoperability towards patient-centered interoperability, by making patients the owners of their medical health records and granting them the decision regarding who can access the data [55]. Table 2 shows a review of blockchain systems in healthcare.

## 3. Our Solution: PatientDataChain

### 3.1. System Overview

**Figure 1:**
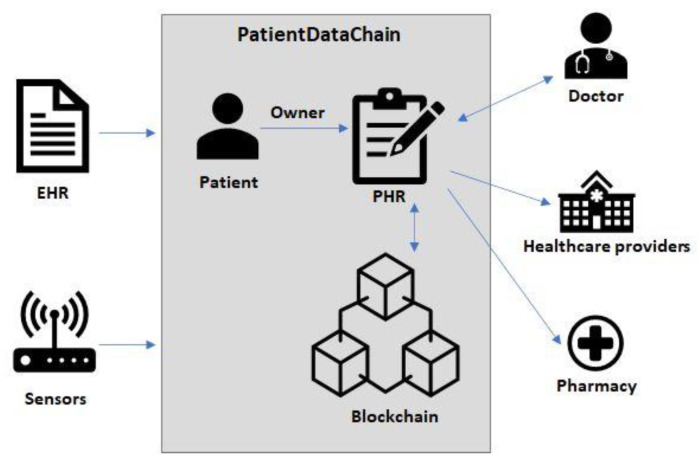
PatientDataChain architectural overview.

Considering the abovementioned issues, our research goal was to propose a decentralized, blockchain-based architecture to integrate patient health records systems, by bringing together all the stakeholders in the healthcare value chain. Our approach was to create a patient-centered model, where patients are the owners of their medical data. Based on a permission-based mechanism, patients were able to give healthcare providers access to their data for a limited period of time. This enabled us to provide an integrated and interoperable approach, that collects and fuses health data from heterogenous sources (Figure 1), such as: the patients’ medical records (e.g., EHR systems), different healthcare providers (laboratories, pharmacies), as well as various sensors from wearable devices (fitness trackers, smartphones, wearable sensors).

The decentralized nature of PatientDataChain, due to the blockchain technology, enhances the proper context to create a novel and improved data sharing and exchange system, which is secure, flexible, and reliable. This approach will bring benefits to data confidentiality and privacy, while providing secure access to patient medical records.

### 3.2. Modex Blockchain Database (BCDB)

Modex Blockchain Database (BCDB) [62] is a hybrid remote data storage system that enables blockchain adoption in enterprise software development and deployment by adding blockchain-based features to existing database engines.

Modex BCDB is designed as a middleware that is installed between clients’ software and their legacy database systems, connecting them to a blockchain engine with minimal effort and without requiring additional training for software developers who use the product, as it has a similar interface to traditional databases. Modex BCDB modifies a set of connectors in order to link the database to a blockchain network, allowing enterprises to build a secure and stable blockchain environment without requiring them to engage in costly research and long development cycles.

It does not eliminate the database component, which is essential for any organization, but adds a set of functionalities to the existing software, making it blockchain-ready, in order to obtain decentralization, data integrity assurance, high availability and scalability, improved security, and a new way to manage business data. Modex BCDB works as a private blockchain network, where each node replicates a blockchain client, a database of the client’s choice, its core functionalities, an administration module, and an API (application programming interface) to allow connections with other systems.

**Figure 2:**
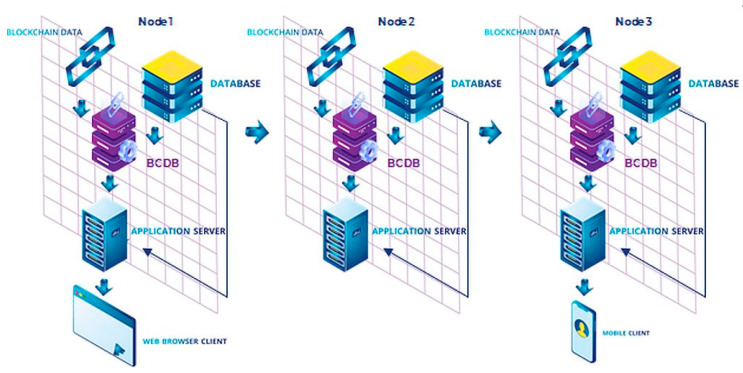
Modex BCDB architecture.

How it works. Modex BCDB combines two architectures (Figure 2): traditional client-server application and the blockchain-based application. Within this reference model, the front-end client communicates with the application server, which is replaced by the business logic. The CRUD (create, read, update, and delete) operations are executed either directly in the database, or through TCP (Transmission Control Protocol) and API calls to the Modex BCDB software solution. Modex BCDB further connects to the database for the data routing process, but it also includes a blockchain component.

**Figure 3:**
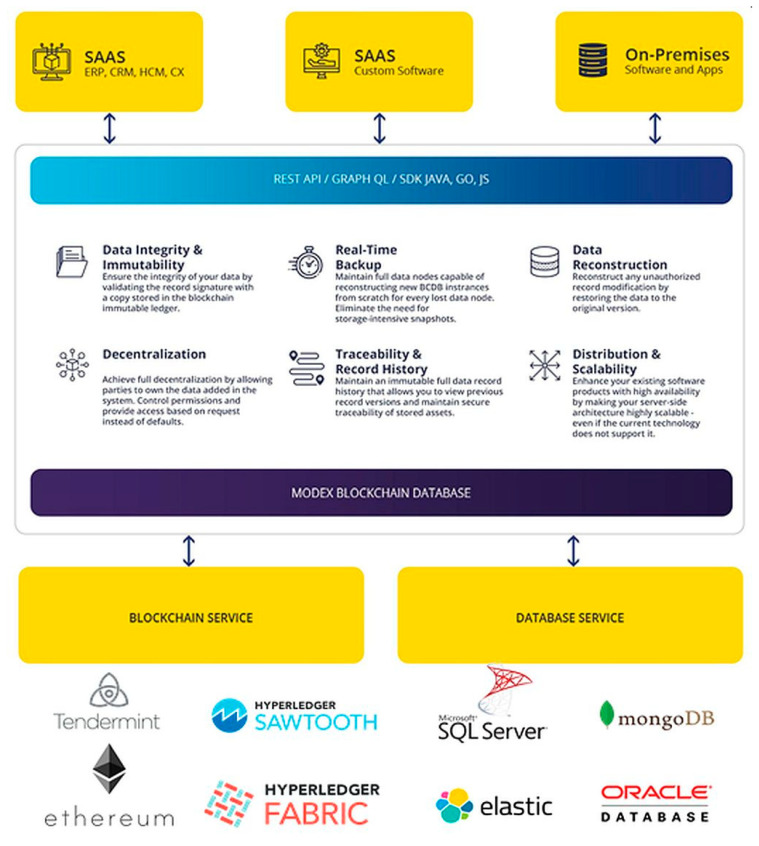
Modex Blockchain Database (BCDB) infrastructure [62]. Reproduced with permission from Modex Ltd.; published on the www.modex.tech website, 2020.

Modex BCDB receives REST (REpresentational State Transfer) API calls directly, using GraphQL or dedicated software development kits (SDK) for Java, Go language, JavaScript. Afterwards, the hash information is extracted and the relevant metadata is computed for each piece of data. The hash of the data is stored in the blockchain service component, and the unmodified data stays in the database service component, together with the computed metadata. The blockchain service can connect to multiple types of blockchain solutions (Tendermint, Hyperledger Sawtooth, Hyperledger Fabric, Ethereum, etc.) as well as multiple types of database solutions, either SQL or NoSQL (MongoDB, SQL Server, Elastic Search, Cassandra, or Oracle DB)—Figure 3. Using the Core API interface, the Modex BCDB Service Processor receives all CRUD operation commands from the business services, and based on configured instructions and security permissions, provides access to the data from the database and the blockchain ledger. The system provides data integrity and immutability, distributing the data across multiple nodes and matching the data in the database with its hash in the blockchain. Because Modex BCDB synchronizes the data using blockchain technology, the data is being backed up on multiple machines in real time, with each insert. In the case of data corruption—if the hash in the blockchain no longer matches the data in the database—the data can be reconstructed from another node where the hash still matches the data. By using blockchain technology we have traceability and a record history, as well as data distribution and decentralization.

### 3.3. System Architecture

**Figure 4:**
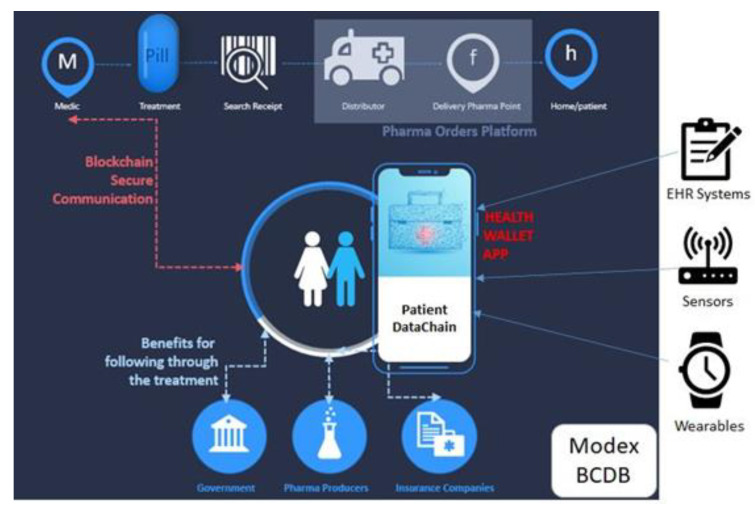
PatientDataChain high-level architecture.

Figure 4 depicts the high-level architecture of the PatientDataChain system and shows its main components and their interrelations.

The system is built over the Modex BCDB blockchain technology, as it provides the main advantage of being a middleware that basically adds a blockchain layer to any existing database. Thus, it is easy to integrate PatientDataChain with any existing EHR systems or existing medical databases, as well as to get data from medical and healthcare sensors and wearables.

The core of PatientDataChain is the Patient Health Wallet app (Figure 4). This is the PHR component of the system, which will unify all the data related to a patient’s health records. The health wallet is the single version of truth, owned by the patient. When a patient has a medical appointment, he will have to grant read/write access for the doctor, so the doctor can see the patient’s medical history and write a new health record after the consultation. When a doctor prescribes a treatment to the patient, the prescription will also be stored in PatientDataChain. The patient can then grant access to pharmacies to see the prescription. The patient will be able to search for the drugs prescribed in the system and make online reservations. Afterwards, he will be notified by the pharmacy when he can pick up his order.

**Figure 5:**
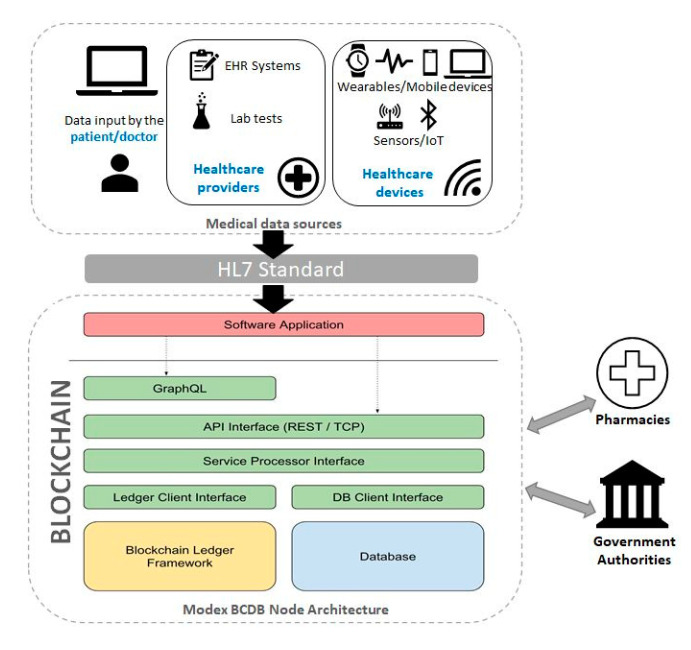
PatientDataChain architectural model.

The diagram depicted in Figure 5 shows the architectural design of the PatientDataChain system. The key element that helps integrate and aggregate the various and heterogeneous medical data sources is the blockchain component, built over the Modex BCDB technology. The network architecture is composed of an array of distributed nodes. Due to the agnostic approach on database engines, the blockchain nodes can be configured with different database connectivity parameters [63].

As can be observed, a node is composed of a multitude of software modules, customized for each application that wants to store data over the blockchain. Each node is responsible for different tasks of the data processing life cycle. The software application client that integrates with the blockchain node provides the business functionality component.

The main tasks handled by the nodes are related to data processing, database connectivity, reading/writing to the blockchain, data synchronization over the networks, permission granting, the software application client. Each of these software modules will be developed according to each application use case and specifications.

The PatientDataChain system components, as shown in Figure 5, are:

**Medical data sources:**

- Data entered by the patient
- Data from existing EHR systems. PatientDataChain connects to EHR systems via APIs, and the middleware capabilities of Modex BCDB allow for the data in the database to be uploaded in the blockchain. Thus, the doctor will continue entering the medical data in his legacy EHR system, and the data will be automatically taken to the blockchain through the API connector.
- Data from various healthcare providers, such as laboratory exams.
- Data from wearables and healthcare tracking devices/sensors (such as fitness trackers or smartwatches).

Medical data are compliant with the HL7 standard.

**Blockchain component**. The interface between the medical data and the blockchain is the software application, namely the Health Wallet app, which is responsible for collecting the data and unifying it. The main component of the blockchain are nodes [63], composed of a multitude of software modules, each responsible with different tasks of the data-processing life cycle, such as: data processing, database connectivity, reading/writing to the blockchain, data synchronization over the networks, permission granting, the software application client. The software application client that integrates with the blockchain node provides the business functionality component. Each node contains the following components: GraphQL, API interface, service processor interface, ledger client interface, database client interface, blockchain ledger framework, database component.

**GraphQL**. PatientDataChain uses GraphQL [64] queries to retrieve the data from the data sources API connectors and store them in the blockchain nodes.

**API Interface**. The integration with client applications is realized through low-level API endpoints. The API interface receives all the CRUD operation commands from users and passes them to the service processor interface. It provides data access through HTTP protocols secured by OAuth2 services. Data manipulation operations are based on a complex hierarchy of configured instructions and security permissions needed to access different data tiers from the database and blockchain ledger. Only full nodes will store a complete copy of the blockchain ledger. To ensure efficiency, and reduce the accumulation of redundant data, partial nodes will synchronize only with data relevant for operations performed at their location.

**Service Processor Interface**. The service processor interface is a key component of the data exchange mechanism, which also manages the data ownership mechanism. System users are the true owners of their data; thus, they are responsible for the information introduced in the blockchain/database. Each user owns a unique private key that can be used to encrypt data both at the database and blockchain level, which will also be used to grant access to third parties to their digital medical records.

**Blockchain Ledger Framework**. The blockchain ledger framework implements the blockchain engine used by the Modex BCDB framework. PatientDataChain uses Tendermint as blockchain engine.

**Database Component**. The database component represents the database engine connected to the Modex BCDB infrastructure. PatientDataChain uses a MongoDB database engine. It would be inefficient and redundant to store all the data on the blockchain. To better handle data with various life cycles, Modex BCDB facilitates traditional database support to store business-relevant data.

Modex BCDB is designed to act as a fusion between a blockchain engine and a traditional database system, to enhance security and data privacy of existing software infrastructures. By combining blockchain technology with a client’s database, the new framework achieves a zero-knowledge proof state, in which data can be provided to third-party actors without revealing any additional information apart from that specific data.

**Figure 6:**
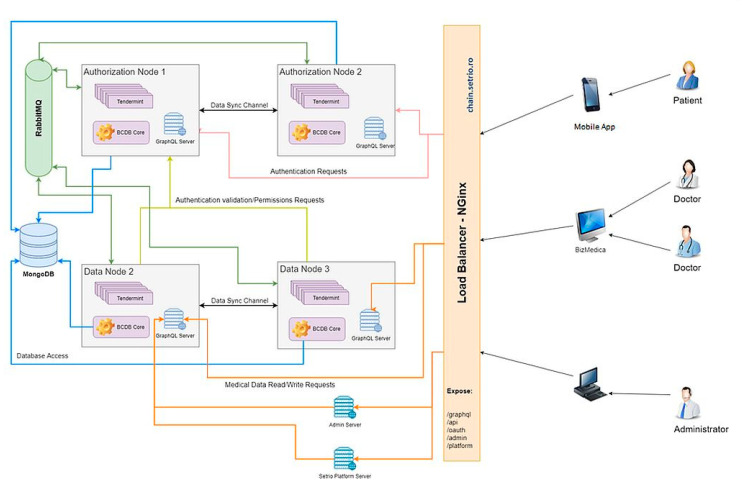
PatientDataChain infrastructure architecture.

Figure 6 depicts the infrastructure architecture for the development of the PatientDataChain system.

The diagram shows the deployment of each node of the blockchain and the data flow within the system. Data is collected from the patients’ mobile app and from the doctors’ web app through the API interface, which uses GraphQL queries to bring the data in the blockchain nodes. The patients’ mobile app, at the moment, contains data entered by the patient and data collected from the Fitbit fitness trackers, via the Fitbit web API. The doctors’ web app is currently integrated with BizMedica as an EHR system. The data is fused based on the unique ID of the patient and saved in the PatientDataChain backend infrastructure. The Tendermint blockchain component of each node will store authenticity elements (timestamp, hash, and encryption key). The medical data will be stored, in an encrypted form, in the MongoDB database and will be accessed on a token-based permission granting basis. The tokens will be generated based on the encryption keys of the patients.

### 3.4. System Development and Functionalities

1. Registration. A patient is allowed to register through the mobile application into the PatientDataChain platform. The user is stored into the Modex BCDB network after the user will confirm the email provided at registration time. A doctor is allowed to register into the PatientDataChain platform after he receives an invitation by email. After registration, the doctor is redirected to the doctor web platform login page. At the first login, the doctor is prompted to complete his/her profile (title, specialties, medical clinics, contact details). The OAuth 2.0 protocol is used for authentication [65] and generates tokens for single sign-on. The token generation is based on the private key of the user.
2. Data fusion. In order to ensure access to an integrated collection of medical data associated to each patient and provide a secure environment for storing personal health records, we developed a data fusion mechanism based on unique identifiers. The system tackles the challenge of data integration in the blockchain by assigning a unique identifier (ID) to each patient. The patient will own his data and can grant access to healthcare providers through a token mechanism, to view the data or to insert new records in his medical file. When a doctor must write on behalf of the patient, first they need to acquire the patient ownerId, generated from the patient identifier created after the first login into the mobile application. The blockchain provides the optimal context to track the unique patient identifier and solving the patient matching issues. In PatientDataChain, the pairing is done using the patientID and ownerID to integrate new data into the patient’s medical history.

**Figure 7:**
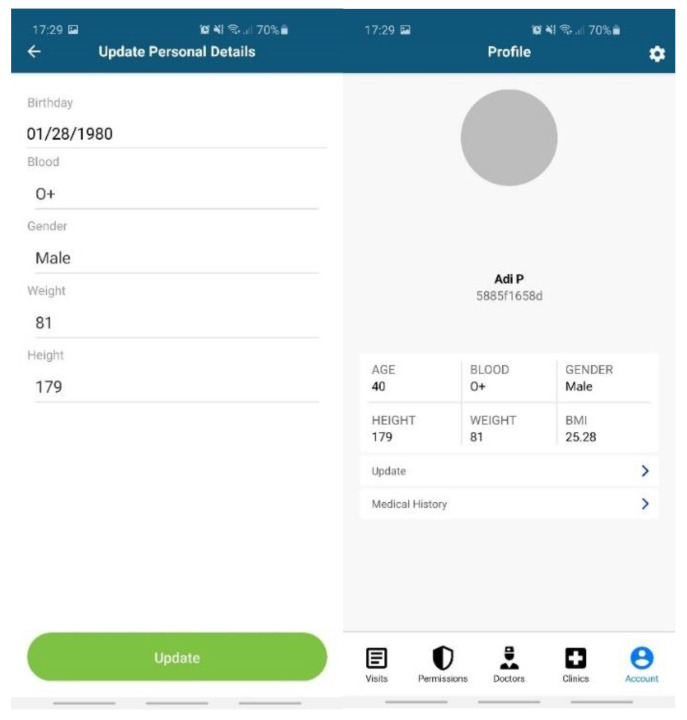
Data entered by the patient in PatientDataChain.

**Figure 8:**
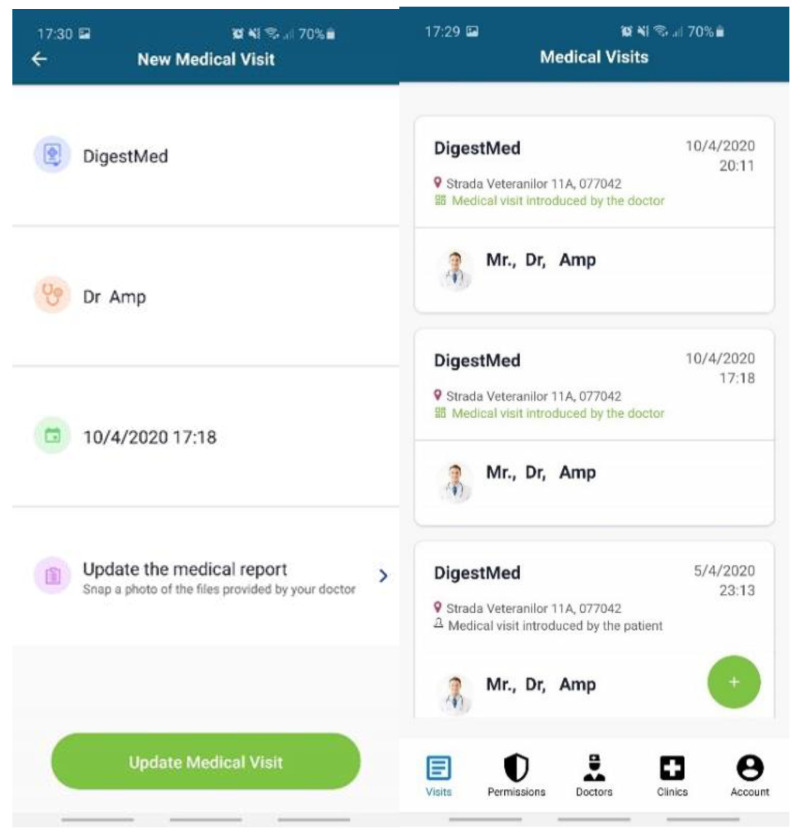
PatientDataChain integration with the EHR system.

The current version of PatientDataChain can collect and unify data from the following sources:

- Data entered by the patient (Figure 7): personal profile (name, age, blood type, gender, height, weight, body mass index (BMI)); allergies; medical history (chronic diseases, surgeries).
- Data from EHR systems. For the moment, we developed a dedicated API based on web services endpoints to connect to Setrio BizMedica [66]—a medical software for managing the activity of individual medical practices and clinics, integrated with the National Healthcare System in Romania. The API interface retrieves data from BizMedica and uses GraphQL queries to insert the data into the Modex BCDB architecture. The blockchain component will store the authenticity elements (timestamp, hash, and encryption key), while the encrypted medical health records will be stored in the MongoDB database, in correlation with the hash from the blockchain. The data from the MongoDB database can only be decrypted with a token generated based on the key in the blockchain. This mechanism enhances security and data protection.
- Doctors and healthcare institutions will continue using their legacy EHR systems, which will be integrated with PatientDataChain through the API interface. The API interface will identify each patient based on his unique ID in PatientDataChain. When a patient visits a doctor, they will grant access to the doctor to view their medical history and insert a new medical record (diagnosis, treatment, lab exams, MRIs, scans, etc.) in PatientDataChain (Figure 8) through a system token generated based on the ownerID field of the patient, which will be valid for a limited time.
- Data from wearables and healthcare tracking sensors, to keep the history of various health indicators as part of the medical history of the patient. Currently, PatientDataChain is integrated with Fitbit devices through the Fitbit web API [67] from Fitbit activity trackers. The data retrieved from the fitness tracker is fused with the medical history of the patient based on the unique ID of the patient. The Get Daily Activity Summary endpoint is used to retrieve a summary and list of a user’s activities and activity log entries for a given day. Other information retrieved from Fitbit devices and integrated into PatientDataChain are related to weight, fat, food logging, heart rate, and sleep.

3. Data ownership. When a user is created, the system generates a unique pair of private/public keys associated with the new user. The keys will be used with RSA 256 encryption to serve several purposes: user authentication, transaction signing, data encryption/decryption. The private/public keys sets are stored on the blockchain, in a dedicated authentication and licensing node. The data ownership is established through the following mechanism: when a patient is logged in and a new record of data needs to be inserted into his medical file, the private key of the patient will be used to encrypt the data. The hash of the data will be stored in the licensing node, whereas the encrypted medical data is stored in the MongoDB database. The private key is what grants a patient ownership of his medical health records.
4. Medical Records Permission Control. Only the patient will be able to see his medical records and, on demand, the patient can give permission to a specific doctor or healthcare provider for a period of seven days. Once the patient has given permission to a doctor to see their medical history, the information will be available for the doctor on the web platform. This permission-granting mechanism is based on the private/public key of the patient. When the patient wants to grant access to a doctor to see the data, a token will be generated based on the public key of the patient, which will allow the doctor to access the medical files and also to insert a new medical visit.

## 4. Validation and Discussion

### 4.1. Methodology

So far, PatientDataChain has been validated through a proof of concept (PoC) implemented in a medical clinic in Bucharest. The clinic reunites practitioners from various specialties and provides medical consultations, as well as laboratory and imagistic investigations. It uses BizMedica as its EHR system, which facilitated the integration with PatientDataChain. We used a dataset of 100 patients and 1144 transactions, over a period of three months. The patients were selected based on their willingness to participate in the pilot project, on condition that they were over 18 years old. When installing the app, the patients gave their consent through an electronic form, through which they acknowledge the beneficiaries of PatientDataChain and the handling of their personal information based on GDPR. The patients involved in the PoC project received the app for free. The data is stored on the blockchain and the patient is the only one who can access the data at any time. Any other medical third party needs permission from the patient for any operation related to the patient’s medical data.

The validation process had two objectives:

- assess the main functionalities of the system and the data exchange features
- analyze the performance of a blockchain-based system and assess the scale-up possibilities

The PoC components run on a Modex BCDB network simulated on a single machine. The doctor web platform and admin web interface were deployed into the same network as Modex BCDB. All services were routed to an external address through an NGinx instance deployed on the same network. The PoC of PatientDataChain focused on the core blockchain-based technological solution (the Health Wallet app); the integration with an existing EHR system (BizMedica) for managing the activity of general practitioners, specialized medical offices, and medical clinics; and the integration with a fitness tracker device (Fitbit Charge), to integrate various healthcare parameters in the patient health records.

Currently, the PatientDataChain system is at TRL5, and our main focus during the PoC validation was to demonstrate the data exchange functionality, in order to ensure that the integrated PHR based on blockchain technology provides the required functionalities for the healthcare ecosystem. Moreover, we analyzed the performance of the system, in order to assess the scale-up potential to a large number of users.

### 4.2. Validation of the System Functionalities

The specific test-case scenarios that have been validated are the following:

- Reading/writing functionalities for the patient/owner of the PHR
- Integration with the EHR system
- Acquiring a short-term user access token for granting access to data
- Getting logged-user details
- Getting doctor profile details
- Getting a patient ownerId based on the patient’s unique identifier
- Writing a medical report and visit on behalf of a patient
- Inserting a medical report
- Inserting a medical visit
- Attaching files to a medical report on behalf of a patient
- Acquiring healthcare data from a fitness tracker

The purpose is to demonstrate that the PHR accurately collects and unifies the patients’ data and that data is correctly exchanged among healthcare providers. In order to validate the above functionalities, we monitored the exchange of messages to make sure that they occur in the right sequence and with the correct content. In what follows, we present a set of relevant sample requests and responses of the system that confirm the demonstration and validation of the PoC.

#### 4.2.1. Getting a Patient OwnerId Using the Patient Identifier

When a doctor must write on behalf of the patient, first you need to acquire the patient ownerId. The patient has to provide the patient identifier from the mobile app generated after the first login into the mobile app.

Sample request:

curl --location --request POST ‘https://chain.setrio.ro/api/data/filter/Identifier?skip=0&limit=1’ \
--header ‘Authorization: Bearer -sm0n18N7MJJzY_og88N95HX8pnndskzGGcw5GaNaU8’ \
--header ‘Content-Type: application/json’ \
--data-raw ‘{
“filter”: {
“patientId”: “fde53c5a11”
}
}’



Sample response:

{
“records”: [
{
“_id”:”59ea2347d6139195b183f5988b4d527710c385c6fed7119699129e94b3769c8f”,
“metadata”: {
“previousBlockId”: “2BBA75BB049B2A0D1F91709903219CB1FD1242178157B2EC6BC28F1CED4306EA”,
“blockId”: “49”,
“id”: “59ea2347d6139195b183f5988b4d527710c385c6fed7119699129e94b3769c8f”,
“blockNum”: “49”,
“version”: 2,
“previousRecordBlockId”: “c4db13e4424fe2865138c0145c7fdd111b728609f4b581cb61ab29a4e8837214”,
“recordId”: “386609bcadcfada54a0c26bdc8f408060d145abc19bff21c2e2f8e4e9d949f8f62bf20”,
“ownerId”: “3866093b83947ddbbf48ec39667fcb6ef2d235992caf5107f0f5e5337fa57810e869bd”,
“status”: “COMMITTED”,
“transactionId”: “59ea2347d6139195b183f5988b4d527710c385c6fed7119699129e94b3769c8f”,
“createdBy”: “3866093b83947ddbbf48ec39667fcb6ef2d235992caf5107f0f5e5337fa57810e869bd”,
“createdAt”: “2020-07-16 14:48:56”,
“modifiedBy”: “3866093b83947ddbbf48ec39667fcb6ef2d235992caf5107f0f5e5337fa57810e869bd”,
“modifiedAt”: “2020-07-16 14:49:06”,
“dataType”: “JSON”,
“nodeId”: “HF-data-01”,
“recordPermission”: “PERMISSIONED”,
“networkIdentifier”: “OPEN”,
“recordSignature”: “5ea3b0b94bd2b8c46f0c97e7fe5d85a4d6dde6f13b652f2173790d0620600d64cea5947839f9caa05ab450a1339a332723c00d668d93c83985f2a167d66b4119”
},
“ownerId”: “3866093b839439a92b368bbedc271cf663c125748982ea4a938096940dfa88e2f49013”,
“patientId”: “fde53c5a11”,
“validated”: “true”
}
]
}

- Writing a medical report and visit on behalf of a patient

For a doctor to write a record on behalf of a patient, the doctor must have the right access to write the record on behalf of the patient. The initial permissions model imported into the system already has this configured, where the doctors group has the right access to write on behalf of the users from the patients group.

- Inserting a medical report

A medical report inserted into the system must be linked to a medical visit record.

Sample request:

curl --location --request POST ‘https://chain.setrio.ro/api/data/MedicalReport?ownerId=3866093b839439a92b368bbedc271cf663c125748982ea4a938096940dfa88e2f49013′ \
--header ‘Authorization: Bearer MNHx6lDQxvzEE-nKchhZmx74L9j2-Q4Z5I6Bh18k_kM’ \
--header ‘Content-Type: application/json’ \
--data-raw ‘{
“diagnosis”: “Hashimoto syndrome”,
“symptoms”: “thyroide is huge”,
“observations”: “take care of you”,
“documents”: [
{
“name”: “medical_report.pdf”,
“serialNo”: “12342r43fr544”,
“description”: “Lorem ipsum”,
“addByUser”: false
},
{
“name”: “reteta.pdf”,
“serialNo”: “xxxx79832793”,
“description”: “Lorem ipsum”,
“addByUser”: false
}
]
}’
		  

Sample response:

{
“transactionId”: “cb2cf0a5e7bd96c706417349448904adc6b119becfc466b369bdd1cae80ca1f6”,
“recordId”: “3866099799650f037ea4694bc0b12c56ab9f5c77728bc3a50056ae865ce2d9d3773513”,
“version”: 1,
“ownerId”: “3866093b839439a92b368bbedc271cf663c125748982ea4a938096940dfa88e2f49013”,
“createdBy”: “3866093b8394889db747715de07b33c196a123ceeaf3fbbc2b94729f9fc2b00b70196e”,
“createdAt”: “2020-07-16 14:57:38”,
“recordPermission”: “PERMISSIONED”,
“nodeId”: “HF-data-01”,
“networkIdentifier”: “OPEN”,
“dataType”: “JSON”,
“systemEntity”: false
}

- Inserting a medical visit

To insert a medical visit record, the following IDs must be used:

- Medical clinic record ID (metadata.recordId of the medical clinic record from the Practice entity)
- Doctor record ID (metadata.recordId of the doctor’s record from the Provider entity)
- Medical report record ID (metadata.recordId of the medical report record from the MedicalReport entity)

Sample request:

curl --location --request POST ‘https://chain.setrio.ro/api/data/MedicalVisit?ownerId=3866093b839439a92b368bbedc271cf663c125748982ea4a938096940dfa88e2f49013′ \
--header ‘Authorization: Bearer MNHx6lDQxvzEE-nKchhZmx74L9j2-Q4Z5I6Bh18k_kM’ \
--header ‘Content-Type: application/json’ \
--data-raw ‘{
“practice”: “386609295f8eb49e42aa726383076fe90dde29303dc344616d7e9dd3cab3e283ffe6ea”,
“provider”: “386609c5a8fdd3f99d3d2f6c26c8dbc09ffb1e1a66cf762e93aa02b6f3a9f6daeeb1e2”,
“startDate”: “2020-02-20T14:00:00”,
“addByUser”: false,
“medicalReport”: “38660997996588813eb31566aff65f7b274338c2f19189224ebc184114851bcb882b8e”
}’
		  

Sample response:

{
“transactionId”: “ca510e0a4de3f1c777ca223f14270231ed8a250756cafbbf9bb04ac62f68653a”,
“recordId”: “3866098bedcf801d694fc2f5c490f0f05091dc78cd54a101f68ebc51dee0923808b373”,
“version”: 1,
“ownerId”: “3866093b839439a92b368bbedc271cf663c125748982ea4a938096940dfa88e2f49013”,
“createdBy”: “3866093b8394889db747715de07b33c196a123ceeaf3fbbc2b94729f9fc2b00b70196e”,
“createdAt”: “2020-07-16 14:58:07”,
“recordPermission”: “PERMISSIONED”,
“nodeId”: “HF-data-01”,
“networkIdentifier”: “OPEN”,
“dataType”: “JSON”,
“systemEntity”: false
}
 

#### 4.2.2. Attaching Files to a Medical Report on Behalf of a Patient

Sample request:

curl --location --request POST ‘https://chain.setrio.ro/api/data/file/upload?ownerId=3866093b839439a92b368bbedc271cf663c125748982ea4a938096940dfa88e2f49013′ \
--header ‘Content-Type: application/json’ \
--header ‘Authorization: Bearer MNHx6lDQxvzEE-nKchhZmx74L9j2-Q4Z5I6Bh18k_kM’ \
--form ‘qqfile =@/home/user/doc.png’ \
--form ‘referenceId=38660997996588813eb31566aff65f7b274338c2f19189224ebc184114851bcb882b8e’ \
--form ‘referenceName=MedicalReport’ \
--form ‘mimeType=image/jpeg’ \
--form ‘qquuid=sdfsdfsdfsds’ \
--form ‘qqfilename=doc1.jpg’
		  

Sample response:

{
“code”: 2000,
“success”: true,
“message”: “File uploaded successfully!”

- Acquiring healthcare data from a fitness tracker

Sample request:

curl --location --request POST ‘https://chain.setrio.ro/api/data/FitbitCharge?ownerId=3866093b839439a92b368bbedc271cf663c125748982ea4a938096940dfa88e2f49013′ \
--header ‘Authorization: Bearer MNHx6lDQxvzEE-nKchhZmx74L9j2-Q4Z5I6Bh18k_kM’ \
--header ‘Content-Type: application/json’ \
-- GET https://api.fitbit.com/1/user/-/activities/heart/date/today/1d.json
}’
		  

Sample response:

{
“transactionId”: “cb2cf0a5e7bd96c706417349448904adc6b119becfc466b369bdd1cae80ca1f6”,
“recordId”: “3866099799650f037ea4694bc0b12c56ab9f5c77728bc3a50056ae865ce2d9d3773513”,
“version”: 1,
“ownerId”: “3866093b839439a92b368bbedc271cf663c125748982ea4a938096940dfa88e2f49013”,
“createdBy”: “3866093b8394889db747715de07b33c196a123ceeaf3fbbc2b94729f9fc2b00b70196e”,
“createdAt”: “2020-07-16 14:57:38”,
“recordPermission”: “PERMISSIONED”,
“nodeId”: “HF-data-01”,
“networkIdentifier”: “OPEN”,
“dataType”: “JSON”,
“systemEntity”: false
“activities-heart”: [
{
“dateTime”: “2020-08-04”,
“value”: {
“customHeartRateZones”: [],
“heartRateZones”: [
{
“caloriesOut”: 273.2109,
“max”: 135,
“min”: 89,
“minutes”: 40,
“name”: “Fat Burn”
},
{
“caloriesOut”: 315.6609,
“max”: 179,
“min”: 128,
“minutes”: 60,
“name”: “Cardio”
},
{
“caloriesOut”: 122,
“max”: 215,
“min”: 175,
“minutes”: 5,
“name”: “Peak”
}
],
“restingHeartRate”: 73
}
}
]
}
		  

Through the PoC conducted, we validated the following functionalities of PatientDataChain:

- The creation of patient and doctor users;
- The registration of medical history by the patient, through the mobile application;
- Integration connector with EHR type applications that can send information in the patient’s digital medical file;
- The web interface through which doctors can view the medical history of patients who grant them the necessary permissions;
- Integration connector with fitness tracker devices from which we collect healthcare data and integrate it into the patients’ PHR;
- Data exchange and immutability granted by the blockchain technology.

### 4.3. Analysis of the System Performance and Scalability

The blockchain infrastructure used in the implementation of the PoC contains six nodes, for which we measured the following parameters for the 1144 transactions performed: transaction duration, total transactions size (TXs_node), total logs, disk used, average transaction size per second (avg_Tx_node).

**Figure 9:**
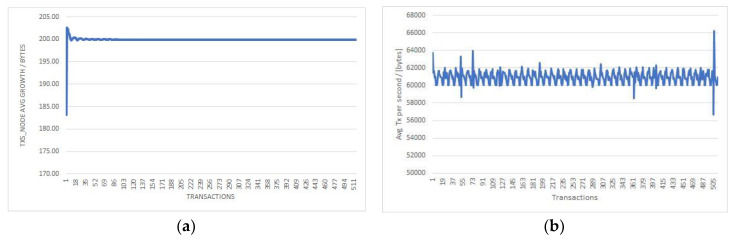
Average transaction size/second (**a**) and average growth in node size (**b**).

Table 3 summarizes the monitored parameters for each node:

Analyzing the results (summarized in Table 3), the “TXs_node average growth/transaction” column and the “Average TX per second” demonstrate a predictable scalability of the PatientDataChain system, in order to accommodate an increasing number of users and transactions. The constant growth in nodes size and average TX (Figure 9) support an increasing amount of transmission capacity.

Moreover, the results of the combined storage model used in PatientDataChain (blockchain and MongoDB) enhance data management in terms of size, enabling the scalability of the system. Throughout the medical history of a patient, PHRs will be composed of many records/datablocks, as well as a substantial number of attachments (laboratory exams results and medical images, scans, magnetic resonance imaging scans (MRIs), etc.) These images usually have large sizes and require bigger storage capabilities. To optimize the traffic over the network, doctors have the option to query the medical investigations over a recent period of time. The query results are sorted chronologically and filtered according to the doctor’s configuration. The same mechanism is used for querying data collected from the fitness trackers, which generate new datasets on a daily basis.

Another potential common issue with healthcare organizations is related to the possibility of having duplicate data entries [68], especially when a patient is registered in two different medical systems that do not interoperate. This aspect is addressed in PatientDataChain through a matching mechanism based on unique IDs. When a new third-party system (EHR or sensor) is integrated with PatientDataChain, the first step is to develop a dedicated API interface that connects it with our PHR system. This interface also contains the matching mechanism, which identifies patients based on their personal data, then queries PatientDataChain, and if the patient is already registered, their unique patientID is retrieved. Their new medical records are then retrieved from the third-party system and stored in PatientDataChain, in the digital medical file of the patient.

As future developments, we plan to:

- Enhance the scalability of the system and the blockchain capabilities to support a larger number of users.
- Provide integration with pharmacies management software, to link the entire medical chain: doctor–patient–pharmacy. This includes the following functionalities:
  - Develop dedicated API interfaces to connect with specific pharmacy management systems.
  - Allow pharmacies registered in the system to display information about the availability of the stock of medicines and their selling prices.
  - Patients can search for the availability of some medicines at the pharmacies in the platform that are located on a certain selected radius.
  - Online reservation of the products found by the search described above and the patient can be notified by the pharmacy when the order is ready for pick-up.
- Automate management of the treatments prescribed to the patient by the doctor—by defining the treatment schemes in the patients’ application and then guiding the patient during the treatment by reminding them of the times when they must take the pills.
- Generate automatic refill orders to pharmacies, for medicines that are part of the treatments and are reaching the minimum stock amount where the refill must be done.
- Implement big data analytics for predictive functionalities.

## 5. Conclusions

This paper presented the design, implementation, and validation of a blockchain-based PHR system that enhances the decentralized storage of medical records and data exchange among the healthcare providers. The system offers patients and doctors an immutable log of healthcare records and improves the patients’ journeys along the health value chain.

The validation proved that PatientDataChain creates the proper context for interoperability and exchange of medical information among health players. The conclusion drawn from the validation study was that the architecture of PatientDataChain, having the patient at its core and putting the patient in control of his/her medical data, leverages a PHR system holding a single and unitary version of the patient’s medical records. After analyzing the performance of the system and its technical capabilities, we concluded that it is scalable to a large number of users. Thus, PatientDataChain mitigates the barriers that the healthcare system is facing because the medical data of a patient are spread among the different medical organizations that the patent interacts with.

## Figures and Tables

**Table 1:** Overview of the interoperability standards in healthcare.

Category	Standard	Short Description
**Vocabulary/terminology standards**	ICD-10 and ICD-11 [13]	Medical classification list of diseases, signs and symptoms, abnormal findings, complaints, social circumstances, and external causes of injury or diseases by the World Health Organization (WHO).
	NDC [14]	List of all drugs manufactured
	SNOMED-CT [15]	Clinical health terminology
	LOINC [16]	A universal code system for identifying health measurements, observations and documents.
**Content standards**	HL7 [17]	Messaging standard that allows the exchange of clinical data between systems
**Transport standards**	DICOM [18]	The standard for the communication and management of medical imaging information.
	FHIR [19]	An HL7 standard for electronic exchange of healthcare information
**Privacy and security standards**	HIPAA [20]	Privacy and security of protected health information (US)
	GDPR [21]	Privacy and security of protected information (Europe)

**Table 2:** Review of blockchain systems in healthcare.

Name	Type of System	Description
MedBlocks [56]	Electronic medical records (EMR)	P2P filesystem made immutable using distributed ledger technology, for storing medical records. Strength: improved consensus mechanism to ensure the network is not overloaded
Secure Health Chain [57]	EMR	Stores electronic personal medical records on a blockchain and ensures that only authorized parties, who have been granted permission, can access data.
MedicalChain [58]	EMR	Decentralized platform that enables storage, exchange and use of medical data. It enables the user to give healthcare professionals access to their personal health data.
Doc.AI [59]	Generate insights from medical data	Enterprise AI platform that uses natural language processing, computer vision and blockchain to generate insights from medical data.
OmniPHR [60]	PHR	Distributed system for storing patient health data. It stores different patient datasets into different blocks on the chain
MIStore [61]	Medical Insurance Storage System	A blockchain-based medical insurance storage system

**Table 3:** Evaluation results.

	Number of Transactions	TXs_Node Average Growth/Transaction (Bytes)	Total Logs Growth/Transaction (Bytes)	Average Disk Used Growth/Transaction (Gigabytes)	Average TX Per Second (Bytes)
Node 1	514	60937	60934	0.22	199.90
Node 2	523	60939	60936	0.21	198.80
Node 3	568	60934	60932	0.19	200.10
Node 4	547	60925	60929	0.17	196.90
Node 5	507	60936	60933	0.21	199.40
Node 6	530	60937	60934	0.22	199.90
